# Effects of endotoxin on pacemaker funny current in HEK 293 cells

**DOI:** 10.1186/cc9663

**Published:** 2011-03-11

**Authors:** VP Papaioannou, A Van Ginneken, AV Verkerk, JB De Bakker

**Affiliations:** 1 Alexandroupolis University Hospital, Alexandroupolis, Greece; 2 Academic Medical Center, Amsterdam, the Netherlands

## Introduction

Different animal *in vitro *studies have concluded that lipopolysaccharide (LPS) can alter electrophysiological properties of ionic currents in cardiac myocytes. There is only one study in the literature that found reduced activation of the pacemaker funny current (IF), encoded by the hyperpolarization-activated cyclic nucleotide modulated-4 (HCN4) gene family, in human atrial cells after administration of LPS.

## Methods

Twenty human embryonic kidney (HEK) 293 cells were transfected with Toll-like receptors-4 (TLR4), CD14 and HCN4 cDNAs and after 24 hours were incubated with 1 μg/ml (10 cells) or 10 μg/ml (10 cells) of LPS (from *Escherichia Coli*; Sigma, St Louis, USA). In addition, 50 pM soluble MD-2 protein was added to the culture medium for enhancing the responsiveness of TLR4 to LPS. Twenty-four hours after LPS addition, electrophysiological recordings were performed at 36°C with the whole-cell patch clamp technique, using an Axopatch 200B amplifier (Molecular Devices, Sunnyvale, CA, USA). IF current properties were measured during 6-second hyperpolarizing steps (range -30 to -120 mV), from a holding potential of -30 mV. Voltage control, data acquisition and analysis were accomplished using custom software.

## Results

Incubation of cells with both 1 and 10 μg/ml LPS was found to significantly impair IF related to controls, by suppressing the current at membrane potentials between -60 and -90 mV and slowing down current activation. Funny current in LPS-treated cells showed more negative half-maximum activation voltage (V_1/2_) values and slope factor (*k*), derived from voltage-dependent activation curves after Boltzmann fitting to experimental data (1 μg/ml: V_1/2 _= -80 ± 3.7 mV and *k *= -14.9 ± 3.4 mV, 10 μg/ml: -96 ± 4.5 and -31.2 ± 6.7, respectively), than the control cells (V_1/2 _= -75 ± 2.8, *k *= 9.7 ± 2.3, *P *< 0.001 for all comparisons). IF current densities between -60 and -90 mV weresignificantly higher in untreated cells (0.67 ± 0.5 pA/pF) than in 1 and 10 μg/ml incubated LPS cells (0.43 ± 0.3 and 0.09 ± 0.05, respectively, *P *< 0.001 for all comparisons).

## Conclusions

In conclusion, this study showed in HEK 293 cells a negative impact of LPS upon activation properties of the pacemaker IF current, confirming findings from previous studies on human atrial cells.
